# Assessment of *Staphylococcus aureus* along milk value chain and its public health importance in Sebeta, central Oromia, Ethiopia

**DOI:** 10.1186/s12866-017-1048-9

**Published:** 2017-06-27

**Authors:** Yodit Ayele, Fanta Desissa Gutema, Bedaso Mamo Edao, Robel Girma, Takele Beyene Tufa, Tariku Jibat Beyene, Fanos Tadesse, Mesula Geloye, Ashenafi Feyisa Beyi

**Affiliations:** 1 Department of Microbiology, Immunology and Veterinary Public health, College of Veterinary Medicine and Agriculture, Addis Ababa University, P.O. Box 34 Bishoftu, Ethiopia; 2 Department of Biomedical Sciences, College of Veterinary Medicine and Agriculture, Addis Ababa University, P.O. Box 34, Bishoftu, Ethiopia; 3 Department of Animal Production Science, College of Veterinary Medicine and Agriculture Addis Ababa University, P.O. Box 34, Bishoftu, Ethiopia

**Keywords:** Antimicrobial resistance, Dairy farms, Milk borne diseases, Milk value chain, Raw milk consumption, *S. aureus*, And staphylococcal food poisoning

## Abstract

**Background:**

*Staphylococcus aureus* is one of the leading causes of gastroenteritis acquired from contaminated foods such as milk and milk products. However, such information is limited in Ethiopia. A cross-sectional study was conducted to assess the contamination of milk with *S. aureus* and knowledge, attitudes and practices (KAP) of actors along the milk value chain in Sebeta, Central Oromia, Ethiopia. A total of 291 samples collected from dairy farms, milk collection centers (MCCs) and processing plant were examined using standard microbiological techniques. The antimicrobial susceptibility profiles of the isolates were also investigated. The KAP of actors in milk value chain were evaluated through a structured questionnaire.

**Results:**

Overall, 23.4% (*n* = 68) of the samples were positive for *S. aureus*. The prevalence of *S. aureus* was 19.6% (95% CI: 14.5–25.6) and 80.0% (95% CI: 14.5–25.6) at farm level and MCCs, respectively. Higher isolation rate was observed in the MCCs (*p* = 0.000) than the farms. The contamination rates of hands of milkers’ and milking buckets with *S. aureus* were 32% and 11.1%, respectively. *S. aureus* was not isolated from pasteurized milk samples. The isolates were found to be resistant to cefoxitin (100%), penicillin G (98.5%), and streptomycin (77.9%). Among 23 interviewed farmers, 35% of them consumed raw milk, none of them wash their hands and 82.6% did not wash udder and teat before milking. Six percent of consumers had the habit of raw milk consumption. Eighty seven percent of dairy farmers and 54% of consumers had no awareness about milk borne diseases and staphylococcal food poisoning.

**Conclusions:**

The study revealed a high prevalence of *S. aureus* along the milk value chain, poor milk handling practices, raw milk consumption behavior, lack of awareness about milk borne diseases and occurrence of antimicrobials resistant *S. aureus*. *S. aureus* seems to pose a public health risk in Sebeta. Authors recommended the urgent need of public awareness creation about the importance of hygienic milk production and proper handling and adequate heat treatment of milk before consumption and further study to assess cost-effective preventive and control options.

## Background

Foodborne diseases are among the most widespread public health problems globally. Food normally becomes a potential source of human infection due to contamination during production, collection, transportation and preparation or during processing [1]. Globally, foodborne illinesses are responsible for an estimated 600 million cases and 420,000 deaths [2]. Among bacteria predominantly incriminated as causes of foodborne diseases, *S. aureus* is one of the leading cause of gastroenteritis, which results from the consumption of food contaminated by staphylococcal enterotoxins [3]. Staphylococcal food poisoning (SFP) is one of the common causes of the foodborne illnesses in many parts of the world. In united states, it was estimated that 240,000 cases of SFP occur each year leading to hospitalization in 1000 cases and death of six people [4]. Similarly, 386 outbreaks in Europe were due to SFP [5]. In France, 300 [6] and in Japan 13,420 [7] cases of food borne outbreak were also reported to be due to SFP. Developing countries bear most of food borne diseases including SFP even though there are no reliable estimates [8].

*Staphylococcus aureus* starts to produce the enterotoxin when the population density in milk reaches about 10^6.5^ cfu/ml [9]. Enterotoxin producing *S. aureus* are dangerous and harmful for the human health, and about 50% of the strains of this pathogen are able to produce enterotoxins that are able to cause food poisoning [10]. A small amount of staphylococcal enterotoxin, ranging from 100 to 200 ng, can cause illness [11].

Milk is highly vulnerable to bacterial contamination, because it supports the growth and multiplication of pathogenic organisms leading to food spoilage, foodborne infection and poisoning [12]. Although pasteurization or boiling of milk is likely to destroy all pathogens including *S. aureus*, public health concern arises when either milk is consumed raw or pasteurization is not efficient [13]. Consumption of raw milk and raw milk products including cheese, cream, butter and yoghurt is common in sub-Saharan Africa including Ethiopia [14]. In Ethiopia, moreover, production and consumption of raw milk and various dairy products often takes place under unsatisfactory hygiene conditions [15]. As a result, the possibility of SFP due to the consumption of dairy products is highly likely [11, 16]. Studies conducted in Ethiopia have indicated the occurrence of *S. aureus* in milk at various points of milk value chain that might be attributed to contamination from mastitic cow, cross-contamination with contaminated milk from infected farm at collection centers, poor handling practices and use of unhygienic equipment [17, 18]. In a previous study, *S. aureus* was identified as a main cause of mastitis in cows in Sebeta, one of the milk shed areas near to Addis Ababa [17]. However, a study about the occurrence of this pathogen along milk value chain at each critical point is absent. To fill the information gap, we assessed the occurrence of *S. aureus* and the antimicrobial sensitivity patterns of the isolates. Besides, the knowledge, attitudes and practices (KAP) of actors along the milk value chain were investigated*.*

## Methods

### Study area and study population

The study was conducted in Sebeta town South West Showa Sebeta district from November 2014 to March 2015. The town is separate district, located in the Oromia Special Zone surrounding Finfinne (Addis Ababa) of the Oromia Region. Sebeta town is located 24 km south west of Addis Ababa at a geographical coordinate of 8°55′N 38° 37′E latitude and 8.917 °N 38.617 °E longitude and an altitude of 2780 meters above sea level. Majority of the dairy farms in the area are kept under small holder intensive farms and animal products, especially dairy products, play a headstone role in household food security both by direct consumption and purchasing of other food items in the area (CSA, 2011). It has an estimated total human population of 49,331of which 24,356 were males and 24,975 were females [19]. There is only one private commercial dairy farm and processing plant that produces milk. The plant also collects milk from nearby smallholder dairy farmers. The plant collects and process about 6300 L milk on average daily. There are no government owned dairy farms and all the farms were owned by smallholder dairy farmers [16].

### Sample size and sampling

Milk samples were taken from individual cows, milk collection centers (MCCs) and processing plant and swab samples were taken from milking buckets and milker’s hands. The samples were assumed to represent the critical points along the milk value chain. The samples were collected and processed during 1 December - 30 April, 2015. About 15 samples were collected per week. The number of cows included in our study was determined using the recommended formula [20] assuming 95% level of confidence (CL), 5% desired level of precision and expected prevalence of 16% [21]. Accordingly, 209 milk samples were collected from individual cows. The dairy cows were randomly selected from 23 dairy farms. Four MCCs were also randomly selected and labeled from the total of 19 MCCs from which 20 milk samples were collected proportionally according to the number of bulk tanks.

Ten pasteurized milk samples were taken from the milk processing plant. In addition, 52 swab samples from 27 milker’s hand and from 25 milk buckets were collected. Overall, 291 samples were included in the study to isolate and identify *S. aureus.* In addition, a total of 110 actors in the milk value chain comprising of 23 dairy farm owners, 1 processing plant worker, 19 milk collectors, 17 hotel/cafe workers and 50 consumers were included in the study. Written consent of the participants were solicited and obtained. The research was conducted after the proposal was presented and approved by the ethical clearance committee of the college.

### Isolation and identification

Cow’s udder and teats were first cleaned with soap and dried using clean towels and then the teats were disinfected with 70% alcohol before sampling. The fore strip milk was discarded and composite milk samples (10 ml) were taken from each cow. Swabs from hands of the milking personnel and milking bucket were collected using sterile, cotton-tipped swabs. After agitating the bulk tank milk, sample was taken from the top of bulk milk using a sanitized dipper from MCCs. Pasteurized milk was purchased from the milk processing plant. Milk samples (10 ml) from individual cows, milk samples from collection centers (10 ml), and 10 packs of pasteurized milk form processing plants were transported to the Microbiology Laboratory of College of Veterinary Medicine and Agriculture of Addis Ababa University, Bishoftu by keeping in icebox containing ice packs. Upon arrival, the collected samples were immediately stored at 4 °C for a maximum of 24 h until culturing the next day.

The bacteriological culture was performed following the standard microbiological technique [22]. A loopful of milk sample using inoculating needle was streaked on sterile 5% sheep blood agar (Oxoid, UK) and swab samples were streaked on blood agar media using cotton applicator and the plates were incubated aerobically at 37 °C and examined after 24–48 h of incubation. The colonies were identified based on morphological characteristics, hemolytic pattern and Gram’s staining reaction. The representative colonies which were positive for Gram’s staining and typical grapes like structure under microscope were further sub-cultured on nutrient agar plates (Oxoid, UK) and incubated at 37 °C for 24 h. pure colonies were preserved and maintained on nutrient slants for further characterization of the isolates. Eventually, identification of the agent was done based on biochemical tests such as catalase, coagulase, mannitol salt agar and purple agar base tests. Samples were considered positive for *S. aureus* when the isolates were catalase and coagulase positive and showed fermentation of mannitol and maltose (strong yellow discoloration of both media).

### Antimicrobial susceptibility test

Antimicrobial susceptibility testing of isolates was performed using the disk diffusion method on Muller-Hinton agar plates as recommended by national committee for clinical laboratory standards [23]. A single colony was selected and emulsified in 3 ml sterile normal saline solution in a sterile test tube. The turbidity of the suspension was then adjusted to the density of a barium chloride standard (0.5 McFarland) in order to standardize the size of inoculum. A sterile cotton swab was dipped into the standardized suspension of the bacterial culture, squeezed against the sides of the test tube to remove the excess fluid and inoculated into Mueller-Hinton agar and allowed to dry the flood. Thereafter, antimicrobial discs [vancomycin [30 μg], penicillin G [10 IU], tetracycline [30 μg], sulphamethoxazole-trimethoprim [25 μg], ciprofloxacin [5 μg], nalidixic acid [30 μg], amoxicillin [10 μg], cefoxitin [30 μg], erythromycin [15 μg], and streptomycin [10 μg]] were placed on the agar with forceps and gently pressed down to ensure contact. The plates were then allowed to stand for 30 min for diffusion of active substance of the agents.

Plates were inverted and incubated at 35-37 °C for 24 h. An inhibition zone diameter of each antimicrobial was then measured and interpreted as ‘resistant’, ‘intermediate,’ and ‘sensitive’ by comparing with recorded diameters of a control organism, ATCC25923 [24].

### Questionnaire survey

A pre-tested structured questionnaire was used to assess the knowledge, attitude and practices of study participants related to handling and consumption of milk in the study area. A total of 110 actors in the milk value chains were interviewed.

### Data management and analysis

Data were entered into excel spread sheet and analyzed using SPSS statistical software version 20. Frequency tables were used to present the data. Chi-square test, Fischer’s exact test and descriptive statistics were used to analyze the data. The significance level was set at α = 0.05.

## Results

### Prevalence of *S. aureus*

Among 291 samples examined, 23.4% (68) were positive. Of this, 19.6% (95% CI: 14.5–25.6%, 41/209) and 80.0% (95%CI: 56.3–94.3%, 16/20) were positive for *S. aureus* at farm and milk collection centers level, respectively. But, *S. aureus* was not isolated from any of the pasteurized milk samples taken from the processing plant. Among the four MCCs, high contamination rate (100%) was observed at milk collection centers labeled as 3 and 7. There was statistically a significant difference in the isolation rate among the milk collection centers (*P* < 0.05) (Table 1). The study has also showed relatively a higher contamination rate of *S. aureus* at MCCs than farm. There was statistically a significant variation (*P* < 0.05).

**Table 1:** Prevalence of *S. aureus* in four milk collection centers in Sebeta, central Oromia, Ethiopia

Milk collection centers	No. of examined samples	No. of positive samples (%)	*P*-value
Center 2	4	2 (50)	<0.05
Center 3	3	3 (100)	
Center 5	6	4 (66.7)	
Center 7	7	7 (100)	
Total	20	16 (80)	

*S. aureus* was found in 32% (*n* = 8) of the total 25 swab samples taken from the hands of milkers. In addition, among the 27 milking bucket swab samples, 11.1% (*n* = 3) yielded *S. aureus*. However, *S. aureus* was not isolated from any of the pasteurized milk samples taken from the processing plant.

### Antimicrobial susceptibility

All the 68 isolates were from (cow milk at farm, milk at MCCs, hand and bucket swabs) of *S. aureus* were tested for antimicrobial susceptibility to 10 selected antibiotics. The isolates were highly susceptible to ciprofloxacin (82.4%), followed by sulphamethoxazole-trimethoprim (67.6%) and nalidixic acid (42.6%), however, they were highly resistant to cefoxitin (100%), penicillin G (98.5%), streptomycin (77.9%), erythromycin (69.1%), and tetracycline (64.7%) (Table 2).

**Table 2:** Antimicrobial susceptibility profile of *S. aureus* isolates according to the types of samples

Antimicrobial	Unit	Type of samples susceptible to different antimicrobial agents											
		Farm milk (*n* = 41) No (%)			Milker’s Hand swabs (*n* = 8) No (%)			Milking bucket swab (*n* = 3) No (%)			MCCs (*n* = 16) No (%)		
		R	I	S	R	I	S	R	I	S	R	I	S
Ciprofloxacin	5 μg	3 (7.3)	5 (12.2)	33 (80.5)	4 (50)	0 (0.0)	4 (50)	0 (0.0)	0 (0.0)	3 (100)	0 (0.0)	0 (0.0)	16 (100)
Nalidixic acid	30 μg	8 (19.5)	23 (56.1)	10 (24.4)	2 (25)	0 (0.0)	6 (75)	0 (0.0)	2 (66.7)	1 (33.3)	2 (12.5)	2 (12.5)	12 (75)
Vancomycin	30 μg	26 (63.4)	0 (0.0)	15 (36.6)	4 (50)	0 (0.0)	4 (50)	2 (66.7)	0 (0.0)	1 (33.3)	12 (75)	0 (0.0)	4 (25)
Penicillin G	10 units	41 (100)	0 (0.0)	0 (0.0)	8 (100)	0 (0.0)	0 (0.0)	2 (66.7)	0 (0.0)	1 (33.3)	16 (100)	0 (0.0)	0 (0.0)
Amoxicillin	30 μg	25 (60.9)	8 (19.5)	8 (19.5)	8 (100)	0 (0.0)	0 (0.0)	2 (66.7)	1 (33.3)	0 (0.0)	12 (75)	2 (12.5)	2 (12.5)
Cefoxitin	30 μg	41 (100)	0 (0.0)	0 (0.0)	8 (100)	0 (0.0)	0 (0.0)	3 (100)	0 (0.0)	0 (0.0)	16 (100)	0 (0.0)	0 (0.0)
Streptomycin	10 μg	36 (87.8)	0 (0.0)	5 (12.2)	4 (50)	4 (50)	0 (0.0)	3 (100)	0 (0.0)	0 (0.0)	10 (62.5)	0 (0.0)	6 (37.5)
Erythromycin	15 μg	28 (68.3)	5 (12.2)	8 (19.5)	4 (50)	0 (0.0)	4 (50)	3 (100)	0 (0.0)	0 (0.0)	12 (75)	2 (12.5)	2 (12.5)
SXT	25 μg	15 (36.6)	0 (0.0)	26 (63.4)	4 (50)	0 (0.0)	4 (50)	1 (33.3)	0 (0.0)	2 (66.7)	2 (12.5)	0 (0.0)	14 (12.5)
Tetracycline	30 μg	28 (68.3)	0 (0.0)	13 (31.7)	8 (100)	0 (0.0)	0 (0.0)	2 (66.7)	0 (0.0)	1 (33.3)	6 (37.5)	0 (0.0)	10 (62.5)

Key: *I* Intermediate, *MCCs* Milk collection centers, *n* number of *S. aureus* isolated, *No* number of *S. aureus* showing resistance, intermediate or susceptible to antimicrobials tested, *R* Resistant, *S* Susceptible, *SXT* Sulphamethoxazole-Trimethoprim

### Knowledge, attitudes and practices

Among the total of 23 interviewed dairy farmers, 35% (*n* = 8) of them consume raw milk. The consumption of raw milk is relatively higher among uneducated dairy farmers (38.5%, 5/13) than those who at least read and write (30%, 3/10). Only 13% of the dairy farmers were aware of the occurrence of foodborne diseases due to raw milk consumption, but none of them have aware of staphylococcal food poisoning associated with consumption of raw milk and milk products. Among 87% (*n* = 20) of farmers who had no aware of food poisoning, 40% (8/20) of them had habit of consuming raw milk. Of the 23 farmers, 60.9%, 21.7% and 17.4% of them practiced cleaning of barn twice, more than two times and once per day, respectively, however, 82.6% (*n* = 19) didn’t wash udder and teat and none of them did wash their hands before milking using anti septic solutions. On the other hand, all of them practiced washing of dairy equipment with hot water and detergents (example soap) before milking. All of the dairy farmers used plastic containers for both milking and milk storage.

Among the 50 consumers, 88% (*n* = 44) of the interviewed consumers drink boiled milk while 12% (*n* = 6) consume raw milk and raw milk products like yogurt. Fifty four percent of them (*n* = 27) had no aware of milk borne disease associated with drinking raw milk and none of the respondents had knowledge about staphylococcal food poisoning. Of the consumers, 58% (*n* = 29), 24% (*n* = 12) and 18% (*n* = 9) of them purchased milk from farms, cafe and from MCCs, respectively. Sixty six percent of them (*n* = 33) used plastic containers while the rest (*n* = 17) used metallic containers to transport milk to their homes. Ten percent of them kept milk in a refrigerator while 90% (*n* = 45) of them kept milk at room temperature. All of the interviewed hotels/ cafes (*n* = 17) purchased raw milk from farms. Among them, 29% (*n* = 5) used metallic container while 71% (*n* = 12) used plastic containers for milk transportation. On the other hand, the respondents (hotels/cafes owners) indicated that they used different methods of quality assessments like boiling (47%) and visualizing and smelling 5 (29.4%) before purchasing milk. The rest 23.6% (*n* = 4) of them directly buy the milk without undertaking quality assessment. Milk was found to be kept in a refrigerator by all hotels/cafes until consumption. Twenty seven percent (27%) of the consumers had no aware of the occurrence of milk borne diseases associated with drinking raw milk and none of the respondents had aware of staphylococcal food poisoning.

At the MCCs, milk from dairy farmers was checked using lactometer reading and an alcohol test for its freshness. Only those milk samples passed the tests were collected from the farmers. Among 19 milk collection centers, 78.9% (*n* = 15) used plastic cans to collect milk. The cans were cleaned with soda ash and hot water at the processing plant and dispatched to the MCCs by vehicles used for transportation of milk to the plant. All the daily collected milk at each milk collection center was used by processing plant to produce pasteurized milk and milk products.

The processing plant, a modern commercial milk processing plant applying milk pasteurization techniques with good manufacturing practices, is located in Sebeta town. Laboratory tests namely petri-film paper test as a rapid microbial quality indicator test was conducted by the plant immediately up on arrival. Besides, milk samples were further subjected to the lactometer reading and alcohol test prior to processing. All the daily milk samples tested and accepted were processed to produce pasteurized milk, cheese and butter. Plastic and aluminum covered cartons were used for packaging of pasteurized milk.

## Discussion

In this study, from 209 lactating cow milk samples subjected to bacteriological examination, 19.6% (41/209) were found to be positive for *S. aureus.* This finding is nearly in agreement with the findings observed in Addis Ababa, 21.13% [25] and 16.2% [21]. However, the result of the present study showed a slight lower contamination rate compared to other works, 29.1% [26], 27% [27], 44% [14] and 75% [28]. This may be attributed to differences in the management practices at farm level.

In the present study, 80% (16/20) of the bulk milk samples from the MCCs were found to be contaminated with *S. aureus*. The results showed a higher contaminated bulk milk of MCCs with *S. aureus* than farm milk. This might be attributed to cross contamination of milk while bulking and poor handling during transportation from farm to collection centers and at milk collection centers [29]. The contamination of *S. aureus* at collection centers was nearly in agreement with the previous work [14, 27] where *S. aureus* was isolated at recovery rate of 75% and 72%, respectively. But, this finding was relatively lower when compared with the report by Addis et al. [29] from which they recovered 46% of *S. aureus* from milk at MCCs. We observed a significant increase of milk contamination with *S. aureus* from farms (19.6% to milk collection centers (80% %); this might be due to mixing of milk from mastitic cows and/or poor handling.

In this study, none of the pasteurized milk samples yielded *S. aureus*. This shows that *S. aureus* were inactivated during pasteurization process and this indicates the absence of post pasteurization contamination and cross contamination during packaging [1]. This is further supported by the fact that *Staphylococcus* species can be easily eliminated from foods by heat treatment [30].

The isolation of *S. aureus* from hands of milker’s and milk buckets were 32% and 11.1%, respectively. These clearly indicated that milk handlers and milk buckets could be the potential sources of contamination of milk with *S. aureus*. The isolation rate from milker’s hand was relatively in agreement with the prevalence rate reported by Deandrade and Zelante [31] and Tondo et al. [32] whose results were 35.7% and 35.2%, respectively. This may be attributed to the fact that staphylococci are ubiquitous organisms and at least 50% of individuals carry the organism in their nasal passages, throat and through coughing or sneezing. They can also contaminate their hands and the variation might be due to differences in milk handling, hand washing and buckets and teats/udder washing practices [33].

The antimicrobial susceptibility tests carried out in this study indicated the occurrence of resistance of *S. aureus* to some of the commonly used antimicrobials. This study presents the sensitivity of the *S. aureus* isolates towards ciprofloxacin (82.4%), SXT (67.6%) and nali﻿dixic acid (42.6%). However, the isolates were found to be highly resistant to cefoxitin (100%) and penicillin G (98.85%). The high resistance pattern of the isolates to penicillin G was relatively similar to the findings reported from different countries: 96.7% [21], 87.2% [34] in Ethiopia and 80% in Sweden [35]. But, it was in contrast to the other findings reported by [36–38] who reported 23% in West India, 57% in Iran and 50% in Finland of resistance to penicillin G, respectively. This is probably due to resistance to β-lactams and frequent use of the antibiotics. Moreover, the present study showed moderate resistance pattern of *S. aureus* to streptomycin (77.9%), erythromycin (69.1%), tetracycline (64.7%) followed by vancomycin (64.7%). The findings are inconsistent with the report of Abera et al. [39] in which 20.6%, 44.8% and 72.4% of resistance level is observed for tetracycline, streptomycin and vancomycin, respectively. This is due to the fact these drugs specifically tetracycline and streptomycin are commonly used in the treatment of infections in the study area. Lacks of stringent regulation and monitoring in the dispensing and use of antimicrobials in the country have also might contribute to the occurrence of high antimicrobial resistance to these drugs.

Antibiotic therapy is an important tool in the treatment of *S. aureus* related infections. However, the misuse or intensive use of antibiotics can lead to the development of resistance among different bacterial strains [40].

The current study revealed that, 82.6% of the farmers didn’t wash udder and teat of the cows before milking and all of them didn’t wash their hands using antiseptics but all washed equipment with hot water and soap (Ajax) before milking. This is in agreement with the report of [41] where many farmers did not sufficiently clean their hands and udder before milking. These practices combined with the observation of the occurrence of *S. aureus* on hands of milkers’ and milk buckets indicated that farmers have no knowledge on the importance of good milking practices in minimizing microbial contamination of milk raising the public health issues. Yet pre-milking udder preparation and employing good milk handling practices play an important role in minimizing contamination at the farm with *S. aureus* [14, 15].

In the present study, the use of plastic containers for milking, storing, collecting and transporting at farm, milk collection centers and by the processing plant was observed. Plastic containers have inherent characteristics that make them unsuitable for milk handling. Plastic containers scratch easily and provide hiding places for bacteria during cleaning and sanitization and poor conductor heat and hence will hinder effective sanitization by heat leading to bacterial contamination of the milk [42–44].

Among the farmers, 35% had a habit of drinking raw milk and 87% of them didn’t have awareness about food borne diseases associated with consumption of raw milk. A study in the USA similarly reported that 42.3% of dairy producers consumed raw milk [45] and other findings also substantiated the raw milk consumption behavior among dairy farmers [11, 16, 17]. Similar to the farmers, 28% of milk consumers drink milk in the form of raw milk and raw milk products like yogurt and 54% of them has no awareness of milk borne diseases. Findings of this study showed that the level of knowledge and awareness of health risks associated with drinking milk by consumers was low when compared with results (20.6%) reported by Karimuribo et al. [46]. Consumers form the last group of the food chain and therefore they are at risk of any malpractice occurring in the chain.

## Conclusions

In conclusion, the present study revealed the occurrence of contamination of milk with *S. aureus* along the milk value chain at farm and milk collection centers. The recovery of the bacteria from hands of milkers’ and milk buckets were found to be the potential sources of milk contamination with this pathogen at the farm. The high prevalence of *S. aureus* at MCCs might indicate cross contamination of milk while bulking and poor handling during transportation from farm to collection centers and at milk collection centers. The study also revealed poor milk handling practices, raw milk consumption behavior, inadequate knowledge of milk borne disease and occurrence of antimicrobials resistant *S. aureus.* In general, the study has revealed the possibility of the public health risk posed by *S. aureus* in Sebeta town. Creation of public awareness about good milk handling practices, milk borne diseases and their prevention is important. In addition monitoring rational use of drugs and periodic assessment of the antimicrobial sensitivity of drugs prior use are recommended. What is more, future studies should consider investigation and designing of cost-effective preventive and control options that would enable to reduce milk contamination by *S. aureus* and thereby the associated public health risks.

## Abbreviations

CFU: Colony-forming units
KAP: Knowledge, attitudes and practices
MCC: Milk collection centers
SFP: Staphylococcal food poisoning
